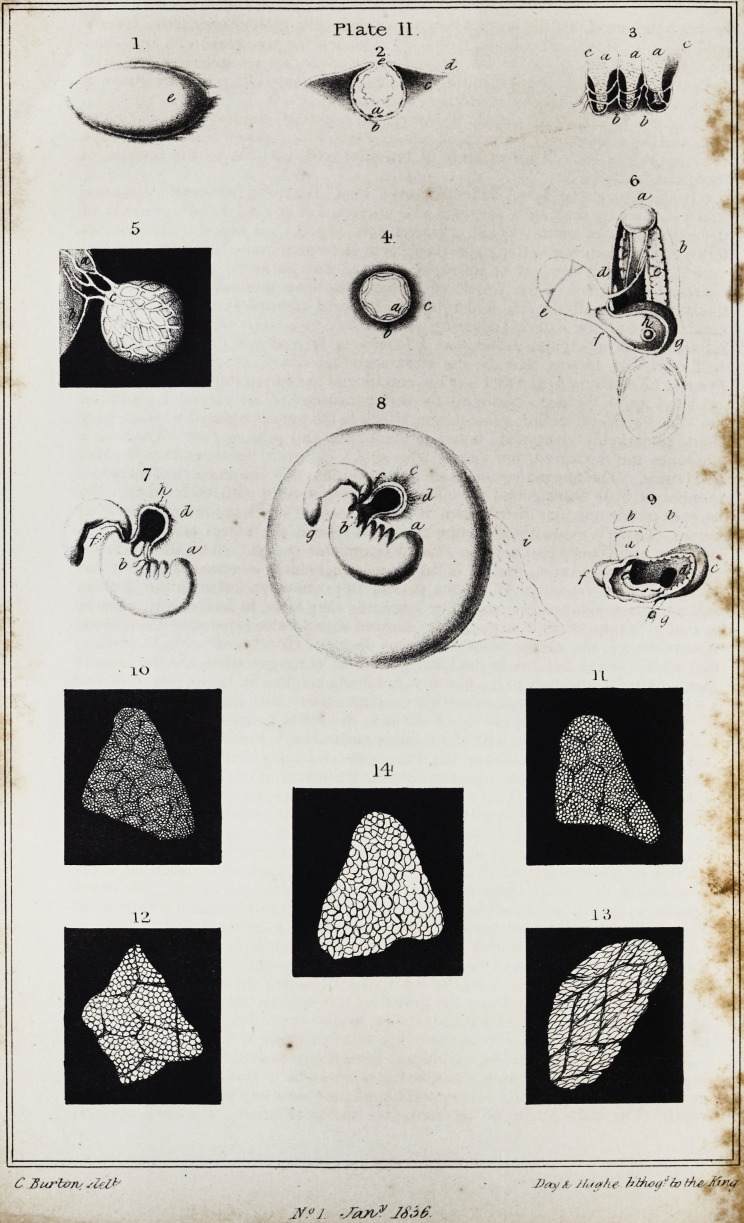# Physiology

**Published:** 1836-01

**Authors:** 


### PHYSIOLOGY.

*On the Early Development of the Human Embryo.* By Prof. von Baer.

[The admirable work of Professor Baer, "*de Genesi hominum et mammalium,*" and the variety of important memoirs with which he has enriched the physiological Journals of the continent, fully entitle him to hold a place in the first rank of physiological enquirers: it gives us sincere pleasure to find that

Plate II.

C. Burton, delt

Day & Haghe lithogs to the King he has announced, in the letter which accompanies the present essay, the speedy publication of his second volume, "*über die Entwickelungsgeschichte;*" and, from the earnest he has given in his present observations, we are indeed justified in anxiously looking for its appearance. A lithographic engraving of illustrations is appended, which we have also added. (Plate II. fig. 1.—9. ]

In figures 1, 2, 3, Pl. II. we see the ovary, the corpus luteum shortly after conception, and the commencing decidua, of a female who had drowned herself eight days after impregnation. The certainty of the date adds no little to the interest of these observations.

"In the ovary (fig. 1, *e*.) I found," says Prof. Baer, "a crescentic entrance into a cavity: by making a perpendicular section, this proved to be the cavity of the corpus luteum, which was not yet developed, (fig. 2.) It was also evident that this corpus luteum was nothing else than the mucous membrane lining the Graafian vesicle, which, on account of its rapid growth, was puckered into rugæ. The external membrane of the vesicle (*b*), which has been unavoidably represented thicker than it really is, had undergone no increase of development, but became continuous with the external covering of the ovary (*d*). The colour of the corpus luteum was of a brighter yellow than I have seen it in any of the mammalia. It will, of course, be seen that *c* is the substance of the ovary, or what I have called germen (Keimlager), and that *e* is the entrance of the corpus luteum, which is not yet filled up." "Upon examining the internal surface of the uterus, I could see the villi (fig. 3,) of its lining membrane, which in the unimpregnated state are very short, remarkably elongated: between these villi, and passing over them, was a substance not organized, but merely effused (*b*), evidently the membrana decidua of Hunter. The uterine vessels were continued into this substance, and formed a number of little loops round the villi; thus anastomosing with each other. On account of this reticular distribution, it was impossible to distinguish arteries from veins. There is evidently the same relation between the uterus and decidua as between an inflamed part and the effused coagulable lymph." Dr. Baer adheres to the general opinion, that it is an exudation, which is connected with the uterus by blood-vessels. At a later period, the connexion between the decidua and mucous membrane becomes more intimate: they form, in fact, one membrane, so that it is impossible to separate the decidua without also separating the mucous membrane of the uterus from its fibrous tissue. He agrees with M. Seiler, that what is called decidua in the latter periods of utero-gestation, has the mucous membrane also attached to it; and that in labour, as also in miscarriages of advanced pregnancy, the mucous membrane comes away with the decidua, but that in the early periods they are quite distinct; the latter being truly an exudation, which but gradually unites with the mucous membrane. Professor Baer considers that the corpus luteum, which in the above case had not yet filled up the cavity of the Graafian vesicle, is evidently produced by a thickening of the inner membrane of the vesicle, (fig. 4.) This drawing represents a perpendicular section of the Graafian vesicle of a female who had had connexion with her lover the day before she drowned herself. "I observed, upon examining the body, a very turgid vesicle, and, on cutting through it, found the internal membrane, which resembles a mucous membrane separated from the external one, evidently thickened, somewhat corrugated, and yellower than in the unimpregnated condition. The corrugation was perhaps produced in making the section; but this could not have taken place without previous detachment, because this inner membrane, before impregnation, is very firmly attached, throughout its whole extent, to the outer covering. Having frequently observed the lining of the Graafian vesicle in animals thickened, and more or less detached from the external coat, before the vesicle had emptied itself, I have no doubt but that the growth of this mucous membrane precedes the opening of the vesicle, and that its opening, as also the discharge of the ovum, are effected by this means. The letters of fig. 4 are the same as those of fig. 2."

The vessels of the vesicula umbilicalis have not been sufficiently demonstrated. Fig. 5 shews a vesicula umbilicalis, which is somewhat separated from its attachments, highly magnified. *b* is a portion of the convexity of the amnion, upon which, at *a*, is the fundus of the diminutive human allantois. *c* is the duct of the vesicula umbilicalis dividing into the two intestinal portions;* and, besides this duct, are two vessels which are distributed upon the vesicula umbilicalis, and form a reticular anastomosis with each other.

The actual open communication between the vesicula umbilicalis and the intestinal tube of the fœtus, has been doubted. The fact, however, may be shown in every healthy embryo at the second month; but the communication is seen most distinctly in those ova where the embryo is attached directly to the membranes. This firm attachment to the membranes Professor Baer considers may be the cause of the early death of the embryo. Fig. 6 is a drawing of such an embryo, which was attached to the amnion by its left side, and where the vesicula umbilicalis is external to the amnion. The development here, as before observed, has been anormal; but, from this reason, the passage from the vesicula umbilicalis into the intestinal canal is shown with such distinctness, that even with the naked eye it cannot be mistaken. The upper portion, with the disproportionately large head, is merely traced in the drawing; but the lower is fully shaded and finished. (*a*) is the curved caudal extremity of the embryo; (*b*) the bladder elongated into a urachus; at (*cc*) are the two false or primordial kidneys, and between them the vertebral column. The posterior portion of the intestine sinks between the bladder and the vertebral column: the part from which it comes is the yelk-bag, or vesicula umbilicalis (*e*), which in this case lay close to the embryo, and was filled with thick vitelline substance, which, although it had been kept for some time in spirit, had not lost its yellow colour: the only effect of the spirit was to coagulate the vitellum into a solid mass, which had broken into several pieces. As no cord has been developed, so also is there no trace of duct to the vesicula umbilicalis, *(ductus vitello-intestinalis;)* the vesicula umbilicalis is merely somewhat elongated, and from its small extremity arises the posterior portion of the intestine; its cavity passing directly into that of the stomach. The cavity *(g)*, which has been laid open in the abdomen of the embryo, is the stomach; above which may not only be distinguished the diaphragm, but also the opening of the œsophagus (*h*).

It is well known that by far the greater number of human ova which we have an opportunity of examining at an early period are anormal; we can only hope to find the contrary in cases of sudden death, but not in abortion. Professor Baer has selected an anormal ovum of this sort in the first month, to show the allantois. "I have," says he, "been able to find the allantois in every human ovum, until the latter part of the second month; but in the natural condition this sac shrivels up as soon as it reaches the external membrane of the ovum, which, on account of this approach of the allantois to the chorion, becomes capable of development, by receiving blood-vessels from it."

Fig. 8 is an embryo in its spherical amnion. This amnion is not two lines in diameter; the embryo is not more than a line. "Even before opening the amnion," says Professor Baer, "I was able, with the naked eye, to distinguish a peculiar appendage. On opening the amnion, I found the embryo attached to its inner surface by an elongation of the amnion (*b*), or short umbilical cord: the shortness of this can scarcely be looked upon as morbid, on account of the embryo being so little developed that the different parts of the head, which is disproportionately larger than the trunk, are scarcely distinguishable, and there are no traces of extremities. The vesicula umbilicalis was situated at this point of attachment, although external to the cavity of the amnion, which latter, I have endeavoured to show by the shading, (fig. 8, *b*,) formed the covering to this intermediate portion of cord and abdomen. From the vesicula umbilicalis I could trace a duct, which was distinctly open, and in part containing vitellum (fig. 7, *h*,) passing into the intestine of the embryo. More anteriorly I could distinguish the vena omphalo-meseraica (*b*); its corresponding artery escaped my notice. The proportions of the vesicula umbilicalis were quite normal, but those of the embryo were not. Its head, even at this early period, is too large in proportion to the body; and this is why the last bronchial fissure lies too far backwards. There are in this case four bronchial fissures similar to that of the mouth (*a*), and so distinctly open that we can see between them on either side, (fig. 7.)

* These intestinal portions are represented rather too thin.

"Having examined the embryo before the amnion was opened, and after being kept for some time in spirit, this appearance cannot be attributed to any previous injury: moreover, the edges of the bronchial processes are well defined, except the last, which passes somewhat indistinctly into the side of the abdomen. The preparation is preserved in the anatomical museum of this place. The chief anormality in this embryo is that the allantois is within the cavity of the amnion. The sausage-like appendage which we see at fig. *g* is a hollow bag, distended with fluid, which comes out from the intestine, or cloaca, of the embryo (*e*), and cannot be anything else than the allantois, which otherwise is found between the amnion and external membrane; nor have I seen it in any other case so full. The drawing before us also shows that the fine membrane, which is frequently found between the amnion and chorion, *(membrana media,)* is not the allantois, as many have supposed, but merely an albuminous fluid between the two membranes; the traces of which may be seen fig. 8, *i*."

Fig. 9 is a sketch of the heart of an embryo, at about the fifth week, laid open, "in which," says Prof. Baer, "I could observe the manner in which the single heart divided itself into two ventricles, with the utmost distinctness. The view of it is from the abdominal side; the ventricle is opened, and the cavity exposed; the heart, therefore, appears much broader than it originally was. The auricle (the division is still so slight that we can only speak of one auricle with a slight contraction in it,) is turned backwards. *d* is the passage of the auricle into the ventricle, which is laid open: from this opening runs a projecting fold (*e*) up to the bulb of the aorta (*a*), which still includes the common origin of the aorta and pulmonary artery: this fold is the commencing septum cordis. The ventricle, therefore, contains but one cavity, but is divided by this septum into two blind sacs, communicating with each other, viz. the future ventricles, the apices of which externally are distinct enough, although the septum is still imperfect. At *g* is seen the ascending cava passing through the diaphragm. At *b*, *b*, are two vessels arising from the aorta at *a*, in order to form the roots of the aorta."

We must here close our notice of this highly interesting and valuable paper. Professor Baer goes on to show that the aorta, at this early period, is formed by the union of two trunks, as in the lower animals; and concludes with some very curious observations on the early development of the pneumogastric nerve, the recurrent branch, &c.

*Siebold's Journal für Gebürtshülfe*, vol. 14, heft 3. *Leipz.* 1835.

---

*The Pulmonary Exhalation experimentally investigated.** By Prof. TIEDEMANN.

[THIS highly interesting memoir was read in August, 1834, before the Hessian Society of Natural History and Medicine, but not published until the present year. We lose no time in laying before the profession its principal contents, as being on matters of the first importance in physiology. We may perhaps be allowed to add, that the gentleman to whom we are indebted for the very faithful transcript from the original, now presented to our readers, himself an experienced and accurate physiologist, was present during the performance of many of the experiments referred to in the memoir. We do not, of course, state this as in any way confirmatory of the truth of the results detailed, for of this the unimpeachable honour of the distinguished experimenter is more than sufficient guarantee; but merely as giving us something of a personal interest in the matter, and accounting for the appearance of some of the notes.]

It is an established fact that man expires water in the form of vapour from the lungs, and that all the warm-blooded animals which breathe by means of lungs exhale watery vapour from the nose. Brodie and Magendie, from the examination of cases of fistulous opening in the trachea below the larynx, denied that it came from the lungs, but asserted that it was formed by the moist mucous membrane lining the nose, throat, &c. This view has been disproved by Paoli† and Regnoli, in

* Die Ausdunstung in den lungen, durch Versuche erläutert.
† Memoria sulla Transpirazione Pulmonare. Pesaro. 1824.

the case of a young female, whose trachea had been opened, and where, at the temperature of 39° Fahr., watery vapour was distinctly expired through the canula.

The views of Lavoisier, that the pulmonary vapour was formed by a combination of the hydrogen in the venous blood with the oxygen of the atmosphere, have been completely overturned by Nysten* and Coutanceau,† who found that, even when animals were made to breathe pure hydrogen or nitrogen, the expired air contained watery vapour. The experiments of Collard de Martigny‡ on nitrogen produced the same result: these last lead us to the conclusion that the watery portion of the expired air is a secretion of the blood during its circulation through the capillary vessels of the membrane lining the air-cells and passage of the lungs: although a portion of it is furnished by the mucus which is secreted by the bronchial vessels, still the chief bulk of it is formed by the numerous ramifications of the pulmonary artery.

Having mentioned the various results of experiments by different physiologists to ascertain the quantity of water given off by the lungs in twenty-four hours, Professor Tiedemann observes, that it is nearly impossible to decide upon the actual quantity of expired vapour, on account of the rapid absorption by the lungs, not only of air containing vapour, but also of vapour which had been already disengaged. The bulk and area of the lungs also varies exceedingly. The condition of the atmospheric air, its density, temperature, and hygrometric state, will doubtless have a considerable influence. The quantity of vapour thrown off by the lungs varies according to the condition of the body; in plethoric healthy individuals a much larger quantity is formed than in those who are emaciated and weakly. Jurine, Lavoisier, and Seguin found that more water and carbonic acid were formed after meals than when the stomach was empty. Magendie observed, in a dog, after he had injected a considerable quantity of warm water into a vein, that the respiration was accelerated, and that a large quantity of water flowed from the mouth. It has also been shown by the experiments of Prout and Fife,§ that much more watery vapour and carbonic acid are formed when a person is awake than when asleep: bodily exertion and mental excitement also increase it. Nysten found that in chronic diseases without febrile excitement, and where the lungs were healthy and the respiration natural, no change was observed; but that, in acute forms, the expired air generally contained more watery vapour and carbonic acid. Jurine ‖ found, in a case of ague, that less carbonic acid was disengaged from the lungs during the cold, than during the hot and sweating stages; in a patient who had been bled to sixteen ounces, less carbonic acid was found in the expired air after the venesection than before. Nysten observed that, in all diseases attended with dyspnœa, the carbonic acid and watery vapour of the lungs were diminished.

According to Collard de Martigny's experiments, the expired vapour of the lungs contains, in 1000 parts, 907 water, 90 carbonic acid, and 3 parts of an animal matter, the nature of which he was unable to determine. That it contains animal matter is proved by the fact that the condensed vapour of the lungs, which has been collected in a cold bottle, if set by for some days in a warm place, will putrefy and disengage an ammoniacal odour; thus showing the presence of a principle containing nitrogen.

The expired matter of the lungs varies exceedingly according to circumstances, and is much affected by the nature of the food or drink: after drinking spirit, or eating onions, &c. the breath is known to smell for several hours; the breath of carnivorous animals is extremely offensive, especially when they have been feeding upon putrid flesh. Orfila¶ gave a dog three drachms of camphor: the respiration became quicker, and the expired air smelt strongly of camphor. He gave a large

* Recherches de Physiologie et de Chimie Pathologique. Paris, 1811, p. 180-231.

† Révision des nouvelles Doctrines Chimico-physiologiques, suivi d'Expériences relatives à la Respiration. Paris, p. 64-296.

‡ Journal Complémentaire des Sciences Méd., Mai et Août, 1830.

§ Annals of Philosophy, vol. ii. p. 328; vol. iv. p. 331.

‖ Senebier, Rapports de l'Air avec les êtres organisés, t. ii. p. 272.

¶ Orfila, Traité des Poisons, tom. ii. p. ii. p. 18.

dog twenty-four grains of phosphorus in one drachm of olive-oil: in the course of a few minutes the animal's breath smelt of phosphorus; vomiting followed, and it died convulsed in the course of four hours. Schubarth* observed that, in animals poisoned with prussic acid, the breath smelt so powerfully of it as to produce headach and vertigo. In horses and dogs to whom camphor, musk, spirit of turpentine, and assafœtida have been given, the smell of these various substances in the breath has been distinctly perceived.

Volatile substances with a strong scent are detected in the breath, if introduced into the circulation in other ways than through the mouth; as by absorption through the rectum, the skin, the serous membranes, or cellular tissue. The members of the Medical Academy at Philadelphia † injected one ounce of tinct. assafœtidæ into the rectum of a cat: the smell of the alcohol was perceived in the expired air in four minutes after the operation; that of the assafœtida in thirty-three minutes. Edwards ‡ remarked that the breath of a young man, to whom he had given half a drachm of camphor in an enema, smelt very strongly of it for a whole day. In a girl to whom Tiedemann gave a decoction of garlic in milk, in the form of enema, on account of ascarides, the breath smelt very distinctly of garlic the next morning. Odoriferous substances, which have been kept for some time in contact with the skin, impart their smell to the expired air. Brandner Stuart § found that his breath smelt strongly of garlic after having applied fresh-bruised garlic to his skin.

A variety of experiments prove that volatile substances, when brought in contact with and absorbed by serous membranes, are discharged in the air expired by the lungs. The physicians of the Medical Academy at Philadelphia injected Tinct. Assafœtidæ into the abdominal cavity of a cat, and in three minutes after, the breath smelt of it. Magendie|| introduced some phosphorus dissolved in oil into the abdominal cavity of a dog: after a few minutes, the animal exhaled a white vapour smelling of phosphorus: the same effect was produced by injecting it into the abdomen. Breschet and Milne Edwards¶ injected a saturated solution of camphor in alcohol into the abdominal cavity of a dog; the smell of the alcohol could be distinguished in three minutes afterward, and that of the camphor in six minutes; which latter continued an hour. From these experiments will be seen the rapidity with which substances absorbed by the stomach or intestines, through the skin or serous membranes, and conveyed into the circulating system, may be discharged from thence with the pulmonary vapour.

Various experiments have been instituted to show that volatile odoriferous substances, introduced directly into the blood, are detected in the expired air. Viborg** injected camphorated spirit of wine into the veins of a horse, and the breath smelt of camphor. Magendie repeated the experiments with the same result: he also injected a solution of phosphorus in oil into the veins of living animals, which exhaled a white vapour smelling strongly of phosphorus. Orfila, Breschet, and Milne Edwards have repeated the same experiments with similar results. Nysten†† found that different gases, which he had injected into the veins of living animals in such small quantities as not to endanger life, were detected in the expired air, and had therefore been removed from the circulation by the pulmonary vapour.

In order to ascertain what substances pass in the form of vapour from the blood

* Bemerkungen über die Wirkungen der Blausäure auf den thierischen Körper, in Hufeland's Journal der Practischen Heilkunde, 1821, Jan. p. 16.

† Philadelphia Journal, No. vi.

‡ Orfila, Traité des Poisons, t. ii. p. ii. p. 20.

§ New-York Med. Repository, vol. i., iii. 1810-1811.

|| Expériences pour servir à l'Histoire de la Transpiration Pulmonaire. Bulletin de la Société Philomatique, 1811, p. 19.

¶ Recherches expérimentales sur l'Exhalation Pulmonaire. Répertoire générale d'Anatomie et de Physiologie Pathologique, t. ii. p. 174, 1826.

** Scheel Geschichte der Transfusion, b. ii. s. 222.

†† Recherches de Physiologie et de Chemie Pathologique. Paris, 1811.

in the lungs, Professor Tiedemann instituted a series of experiments on dogs. Having exposed the femoral vein, and introduced a small injecting pipe with a stop-cock, he was enabled to ascertain how long the substance was before it passed off by the lungs, and how long it continued to do so.

He injected one drachm of the expressed juice of pounded garlic into a vein in the thigh of a middle-sized dog: in the space of three seconds after the injection the breath smelt powerfully of garlic, and the respiration was quicker and deeper; the smell of garlic was quite distinct in the expired air for two hours after. The experiments appeared to have no injurious effects whatever on the animal.

He injected an ounce of spirit of wine into a vein in the thigh of a middle-sized dog. The injection was scarcely over before the delicate, almost etherial, vapour of the alcohol was perfectly distinct.* Respiration very quick, pulse extremely rapid, pupils dilated: the animal lay comatose, and respiration became unequal, ceased, and death followed in ten minutes: in examining the body after death, the alcoholic smell was distinct in the vapour arising from the peritoneal, pleural, and pericardiac cavities; the heart contracted feebly from mechanical irritation; the blood on the right side was dark red, somewhat coagulated, and smelt (as did also that of the jugulars) of alcohol. The blood of the left side was of a bright red, and smelt much stronger of alcohol. On opening the head and vertebral canal, the smell of alcohol was perceptible as in cases of death from intoxication. The cerebral vessels were much injected. Death was produced by destroying the action of the brain and nervous system.

Professor Tiedemann injected half an ounce of camphorated spirit of wine, at twenty minutes past eleven, into a vein in the thigh of a large stout butcher's dog, which had been fed on bread at eight the same morning. In sixteen seconds after the operation, the smell of camphor was perceptible in the expired air, and rapidly increased; respiration much hurried, breathing deep, vehement, and irregular: in the course of a minute, the animal howled, and this was followed by most violent convulsions, viz. opisthotonos, pupils greatly dilated. In order to quiet these effects of the camphor, he injected into the vein half an ounce of cold vinegar, a well-known remedy in poisoning by camphor: the convulsions ceased, and the animal became quiet; the respiration was more regular, although still quick; the pupils continued dilated. Having withdrawn the tube, and tied the vein, the dog was removed from the table. At first it staggered a good deal, and then remained standing still. At twelve o'clock it ate some bread and milk greedily; the pupils gradually contracted; the smell of the camphor continued for some time, growing weaker, and the next day the animal was well and lively.

At thirty-two minutes past eleven, five grains of the best musk, finely diffused in two drachms of water, were injected into a vein in the thigh of a small terrier bitch. The animal became restless instantly after the operation; it breathed quicker and deeper, and cried somewhat. The smell of musk was distinctly perceptible in the expired air. In the course of a few minutes it became comatose, and fell into a species of catalepsy. When removed from the operating table, it stood motionless on its feet, hanging the head down and resting it on the nose.† When the feet were moved, it remained in the same posture in which it was put. The respiration became again regular; the action of the heart was not quickened; it beat seventy in the minute, as before the injection; pupils dilated. In the course of ten minutes

* The writer of this article was stationed at the animal's nose, in order to detect the first traces of alcoholic vapour. He watched the piston of the syringe, as it gradually descended along the cylinder in Professor Tiedemann's hand: it had barely ceased to move before he perceived the peculiar smell, as described above.

† The effects of the musk in this experiment reminded us strongly of some experiments performed on animals, some years ago, by Flourens, to determine the precise functions of the different portions of the nervous system. He removed the cerebrum of an animal: it immediately ceased to exhibit voluntary motions, whether of mammalia or aves. It remained standing, as if in a deep sleep; if pushed, it walked; if he threw the bird into the air, it flew. The animals no longer moved from their own impulse. They retained their power undiminished, but the inward power of determining to act failed entirely.—Rev.

the pupils contracted. The animal moved itself slowly, and evacuated solid fæces. At twelve o'clock it lay down, and fell into a deep sopor, lying on its side with outstretched feet and the eyes half open; the pupils were contracted, the muscles stiff; occasionally, slight convulsive motions appeared; the hind feet were spasmodically contracted. At one o'clock thin mucous fæces flowed from the rectum; the expired air no longer smelt of musk; the animal heat was not perceptibly increased. When the animal was raised and placed upon its feet, it sank down immediately, and could not be roused from its state of sopor. Every now and then slight tetanic actions were observable. At two o'clock it again passed fluid fæces, with a good deal of black blood intermixed: the convulsions became less frequent; the sopor and the discharge of blood from the anus continued; respiration became irregular; the pulse ceased, and it died during the night.

On examining the body the next morning, the muscles were rigid; the veins of the abdomen were distended with dark-coloured blood; the whole intestinal canal was very red; the mucous membrane of the stomach had a reddish tinge; that of the whole intestinal canal was of a dark red; the canal also contained a quantity of effused dark blood in its lower part, mixed with blood, the vessels of the liver and spleen were gorged with dark blood; the bile in the gall bladder was unchanged; the cavities of the heart and large vessels contained dark blood; there was no peculiar change in the lungs, nor in the brain and spinal marrow, except that the veins of the latter contained a large quantity of blood. It was remarkable, that in no part of the body was the slightest smell of musk perceptible: it must either have been entirely excreted, or have undergone some change and decomposition.

Professor Tiedemann observes, that the death of the animal appeared owing to a change produced by the musk in the blood, which had rendered it incapable of maintaining the activity of the nervous system. Nature here had evidently made a powerful attempt to discharge this substance from the blood, partly by the pulmonary vapour, and partly by the mucous membrane of the intestinal canal.*

Two drachms of sulphuret of carbon were injected into a vein in the thigh of a large dog. The injection was scarcely over before it was smelt most powerfully in the expired air; the respiration was very hurried, became soon irregular, intermitting, and ceased. The animal died suddenly, after a short but violent extension of the limbs. The diaphragm no longer contracted on stimulating the phrenic nerves; every cavity of the heart was strongly distended with blood; irritation produced very faint contraction. The blood of both sides of the heart was of a dark red, with no smell of the sulphuret of carbon; it was converted into a homogeneous mass without coagulating. The lungs were covered with large dark red spots, and appeared, hard as if hepatized.

Five grains of phosphorus, dissolved in two drachms of oil, were injected into a vein in the thigh of a hound at ten A.M.: the instant the injection was over, the animal exhaled clouds of a dense white phosphorous vapour from its nostrils and mouth:† it howled, the respiration became hurried, and the heart more active. In half an hour afterward, the expired air still smelt of phosphorus; the respiration became very labouring; the circulation slower, irregular, and the dog died at half-past one. On examining the body, the trachea and its ramifications were found filled with white foam, mixed with streaks of blood; the lungs were of a dark-red colour, covered with red spots, inflamed, aud very dense. The cavities on both sides of the heart were filled with dark coagulated blood, which smelt of phosphorus.

"I have repeated this experiment," (says Professor Tiedemann,) "several times. If it be performed in a dark place, the expired vapour becomes luminous, and the animal appears as if it were breathing fire. If we inject a considerable quantity of phosphorated oil, death follows very rapidly, from the inflammation of the lungs, and consequent obstruction to the breathing."

* The appearance of the mucous membrane of the whole intestinal canal was singular: it was in the highest state of dark-red congestion, and had almost a pulpy appearance.—REV.

† The animal's head was for a moment almost concealed by the dense fumes of phosphorous acid, which streamed profusely from his nostrils and jaws.—REV.

From these experiments and observations, it appears evident that the volatile substances introduced into the circulating system of animals are thrown off with the greatest rapidity from the minute ramifications of the pulmonary artery in the cells of the lungs, and thence removed in the expired air, in which they become easily perceptible to the olfactory organs.

The function of the lungs does not only consist in effecting an important and absolutely necessary exchange between the component parts of the inspired air and those of the dark red venous blood mixed with chyle and lymph, during which the oxygen of the expired air combines with the blood, and the carbonic acid is thrown off, producing the bright red arterial blood; but, besides this, the lungs appear to act as a genuine excretory organ for the venous blood. Volatile unassimilable substances, which have been conveyed into the blood from the food, which cannot serve in forming arterial blood, and are capable of evaporation, are thrown off in the cells and bronchi of the lungs, and removed with the expired air. Thus, the lungs assist in preparing the arterial blood from the alimentary matters which have been digested and carried into the circulating system, and impart to them such combinations and properties as fit them for the office of reproduction, and render the blood conveyed to every organ capable of repairing those changes in the structure and organs of the animal body which have taken place in the performance of their various functions.

In this manner the lungs play a most important part in the process of assimilation and nutrition, not only as the peculiar organ of respiration, but also as a means for the excretion of those volatile substances, and thus enabling the animal functions to preserve their peculiar constitution and qualities, so necessary for the continuance of life. Hence we can understand how variable the pulmonary vapour must be according to the nature of the food, drink, and medicines, which have been taken, and also according to the condition of the vital actions which are accompanied by constant changes in the structure of the different organs. Among other circumstances under which the expired air is remarkably changed, is a state of great hunger. Many physiologists, who have made experiments on animals to ascertain the effects of starvation, have remarked the highly offensive, almost putrid, smell of the breath under these circumstances. It is a well known fact, that the breath of a person who has been some hours without food smells disagreeably. The smell of the pulmonary vapour varies at different ages: in children and youth, the breath has no peculiar smell; but, in advanced age, it has frequently an offensive odour. In females, during the catamenial periods, it has a disagreeable sweetish smell; during pregnancy, also, it has a different smell from that which it has in the unimpregnated state; and, after labour, it has a peculiar milky smell. The smell of the pulmonary vapour varies remarkably in disease: in the advanced stages of phthisis, it is very disagreeable, even putrid and ammoniacal; in malignant putrid fevers, where the blood has been much diseased, the breath is frequently quite fœtid: the breath of rickety, scrofulous children is known to have a sour smell. The pulmonary vapour undergoes a remarkable change when other excretions are suppressed: thus, it smells strongly urinous in suppression of urine. There can be little doubt but that, in certain forms of disease, a variety of miasmata and contagious principles are thrown off by the blood-vessels of the lungs, and that many of those remarkable changes called crises, which are occasionally observed in fevers, are effected in this way.

[Professor Tiedemann's observations present a rich field of enquiry, not only in physiology, but also in pathology and therapeutics. We have given the chief of the references which he has made to different authors in notes, as they may prove useful to those engaged in the same pursuits.]

*Zeitschrift für Physiologie*, vol. V. Part 2. *Heidelberg*, 1835.

---

### *Temperature of the Body in Diseases.*

MM. Becquerel and Breschet have made the following experiments on the temperature of various parts of the body in disease. We have given the temperatures by the Fahrenheit scale, although the Centigrade was employed by the experimenters.

No. 1. Man, æt. 37. Typhoid fever, complicated with bronchitis. Pulse 116.

| | | |
|---|---|---|
| Temperature of the biceps muscle of the arm | . | 102 Fahr. |
| mouth | . . . | 103.37 |

2. Man, æt. 24. Enteritis with bronchitis. Pulse 116.

| | | |
|---|---|---|
| Temperature of the biceps muscle | . . | 103.10 |

3. Young scrofulous girl, during a well-marked febrile attack.

| | | |
|---|---|---|
| Temperature of the mouth | . . .. | 99.10 |
| an inflamed strumous tumour on the neck, | | 104 |
| a phlegmonous tumour in the cellular tissue, | | 104 |

4. Woman, æt. 30. Tumour of the same nature.

| | | |
|---|---|---|
| Temperature of the mouth | . . . | 97.15 |
| a tumour on the neck | . . | 99.10 |
| biceps muscle | . . . | 98.50 |
| the adjacent cellular tissue | . | 95 |

5. Woman. Cancer of the breast.

| | | |
|---|---|---|
| Temperature of the mouth | . . . | 98 |
| the cancer | . . . | 98 |
| fungous granulations | . . | 98 |
| biceps muscle | . . . | 98 |

6. Young man, in a marked febrile attack.

| | | |
|---|---|---|
| Temperature of biceps muscle | . . . | 102.10 |

7. Young man, with scrofulous caries of the bones of the foot.

| | | |
|---|---|---|
| Temperature of the mouth | . , . | 97.70 |
| the biceps muscle | . . . | 99.50 |
| the wound | . . . | 89.60 |

8. Man, æt. 19. Hemiplegia of the left side, with gangrena senilis commencing in the legs.

| | | |
|---|---|---|
| Temperature of the biceps muscle of the arm of the healthy side | . . . | 97.70 |
| diseased side | . . . | 98 |
| mouth | . . . | 97.52 |
| calf of the sound side | . . | 98 |
| paralysed side | . | 98 |

9. Female, æt. 45. Numbness and acute pains in the lower limbs, following paraplegia. Pulse, 84.

| | | |
|---|---|---|
| Temperature of the biceps muscle | . . | 98.85 |
| adductors of the thigh | . . | 99.23 |

10. Man, æt. 60. Mercurial tremor.

| | | |
|---|---|---|
| Temperature of the right biceps muscle, where there was the greatest tremor | . . | 98.67 |
| left side | . . . | 98.87 |

11. Abdominal dropsy, with affection of the heart.

| | | |
|---|---|---|
| Temperature of the biceps muscle | . . . | 98.69 |
| liquid contained in the abdomen | | 98.33 |

12. Man, with confluent small-pox. Some minutes before death, the pulse 144, and very weak.

| | | |
|---|---|---|
| Temperature of biceps | . . . . | 96.53 |
| the hand | . . . . | 89.60 |

The conclusions derived from these facts are, (considering the natural temperature of the muscles as about 96.4 F.):

1. That the febrile condition may increase the temperature 5°.2 F.

2. That very inflamed scrofulous tumours do not produce a higher increase of temperature than the febrile condition: (the purulent parts, it should be remarked, do not share in this increase.)

3. In cancer, there is a slight decrease in temperature.

4. In paralysis, there is no sensible difference in the temperature of the sound and the paralysed limbs.

N B. The biceps muscle of the arm was made use of in these experiments.

*Séance de l'Académie Royale des Sciences,* 10 Août, 1835.

---

## Figures and Tables

**Plate II f1:**